# Molecular, Metabolic, and Nutritional Changes after Metabolic Surgery in Obese Diabetic Patients (MoMen): A Protocol for a Multicenter Prospective Cohort Study

**DOI:** 10.3390/metabo13030413

**Published:** 2023-03-10

**Authors:** Mansor Fazliana, Zubaidah Nor Hanipah, Barakatun Nisak Mohd Yusof, Nur Azlin Zainal Abidin, You Zhuan Tan, Farah Huda Mohkiar, Ahmad Zamri Liyana, Mohd Nawi Mohd Naeem, Norazlan Mohmad Misnan, Haron Ahmad, Mohd Shazli Draman, Poh Yue Tsen, Shu Yu Lim, Tikfu Gee

**Affiliations:** 1 Nutrition, Metabolism and Cardiovascular Research Centre, Institute for Medical Research, National Institutes of Health, Ministry of Health Malaysia, Shah Alam 40170, Selangor, Malaysia; 2 Department of Surgery, Faculty of Medicine and Health Sciences, University Putra Malaysia, Serdang 43400, Selangor, Malaysia; 3 Herbal Medicine Research Centre, Institute for Medical Research, National Institutes of Health, Ministry of Health Malaysia, Shah Alam 40170, Selangor, Malaysia; 4 KPJ Damansara Specialist Hospital, 119, Jalan SS 20/10, Petaling Jaya 47400, Selangor, Malaysia; 5 Sunway Medical Centre, No. 5 Jalan Lagoon Selatan, Bandar Sunway 47500, Selangor, Malaysia; 6 iHeal Medical Centre, Menara IGB, Mid Valley City, Lingkaran Syed Putra, Kuala Lumpur 59200, Malaysia; 7 Sunway Velocity Medical Centre, Lingkaran SV2, Sunway Velocity, Kuala Lumpur 55100, Malaysia

**Keywords:** type 2 diabetes, obesity, bariatric surgery or metabolic surgery, diabetes remission, metabolomics, microRNA, hormones, adipokines, body composition, nutrition

## Abstract

Metabolic surgery is an essential option in the treatment of obese patients with type 2 diabetes (T2D). Despite its known advantages, this surgery still needs to be introduced in Malaysia. In this prospective study, the pathophysiological mechanisms at the molecular level will be studied and the metabolomics pathways of diabetes remission will be explored. The present study aims to evaluate the changes in the anthropometric measurements, body composition, phase angle, diet intake, biochemistry parameters, adipokines, microRNA, and metabolomics, both pre- and post-surgery, among obese diabetic patients in Malaysia. This is a multicenter prospective cohort study that will involve obese patients (*n* = 102) with a body mass index (BMI) of ≥25 kg/m^2^ (Asian BMI categories: WHO/IASO/IOTF, 2000) who will undergo metabolic surgery. They will be categorized into three groups: non-diabetes, prediabetes, and diabetes. Their body composition will be measured using a bioimpedance analyzer (BIA). The phase angle (PhA) data will be analyzed. Venous blood will be collected from each patient for glycated hemoglobin (HbA1c), lipids, liver, renal profile, hormones, adipokines, and molecular and metabolomics analyses. The serum microRNA will be measured. A gene expression study of the adipose tissue of different groups will be conducted to compare the groups. The relationship between the 1HNMR-metabolic fingerprint and the patients’ lifestyles and dietary practices will be determined. The factors responsible for the excellent remission of T2D will be explored in this study.

## 1. Introduction

Bariatric surgery is now also known as metabolic surgery. It is considered a well-supported treatment for type 2 diabetes (T2D) in obese patients, and has been approved by international medical and diabetes organizations as it improves glucose homeostasis [1]. According to the Malaysia National Health and Morbidity Survey (NHMS) 2019, the prevalence of diabetes in adults was 18.3% in 2019 [2]. The prevalence of obesity among Malaysian adults was 20.1%, based on the findings of the same study [3]. While lifestyle modifications, such as dietary and physical activities, should be the first treatment recommendation, metabolic surgery is an option for eligible patients. Uncontrolled T2D may lead to several acute and chronic complications [4]. Early action in tackling this metabolic risk should be considered a priority, as only some people adhere to lifestyle interventions and achieve long-term glycemic control and weight loss [5]. However, there are risks and complications associated with metabolic surgeries, which range from immediate, surgical-related complications to mortality [6]. Metabolic surgery’s overall physiological and behavioral effects do not involve a single mechanism, but multiple signaling pathways [7]. Understanding the mechanism of the effect of metabolic surgeries on diabetes could lead to precision medicine. Therefore, we want to explore this intervention’s molecular and metabolomics effects in the Malaysian context.

Compared with Europeans, Asians develop T2D with insignificant obesity [8]. A meta-analysis demonstrated a higher diabetes remission rate in the Asian population in comparison with the non-Asian population after two years [9].

Sleeve gastrectomies (SGs) and Roux-en-Y gastric bypasses (RYGBs) are the most common types of metabolic surgery. These surgeries alter the gastrointestinal anatomy, as well as the nutrient flow. These changes in the gastrointestinal signals result in weight loss and metabolic improvements [10].

Adipose tissue dysfunctionality could result from increased visceral fat accumulation, leading to the onset of obesity-related comorbidities, and it is associated with the development of T2D [11]. The biomarkers released by adipose tissues can be beneficial or harmful when acting through the pathways of the autocrine, endocrine, and paracrine systems [12]. Dysfunctional adipose tissue is the main source of circulating microRNAs (miRNAs). Various metabolic processes involve these miRNAs in weight homeostasis [13]. The differential expression of circulating miRNAs, both before and after various weight loss interventions, identified potential weight-loss-related candidate biomarkers [14]. The changes in circulating miRNA levels induced by weight loss interventions, including bariatric surgery, indicate a role for these miRNAs as mediators of disease pathogenesis [15].

Although metabolic surgery is common in developed countries, it has only started to gain recognition in Malaysia over the past few years. The serum miRNA biomarkers of sustained weight loss following metabolic surgery are not yet well-established [16]. It will be exciting to study these potential biomarkers in our population.

A newly explored area, the phase angle (PhA), is a potential predictor of postoperative weight loss after metabolic surgery [17]. Generally, the PhA, which is measured by bioelectrical impedance analysis (BIA), reflects the interrelated ratio of the extracellular to intracellular fluid volume [18]. In obese populations, an increase in excess adipose tissue affects health-related physical fitness. This can affect the PhA, which is a promising cell health indicator, and the integrity indicator [19].

This multicenter prospective cohort study aims to study the effects of metabolic surgery on obese individuals with T2D using a molecular and metabolomic approach and nuclear magnetic resonance (NMR). Additionally, this study aims to evaluate the role of the PhA in diabetes remission and weight loss after metabolic surgery.

## 2. Methods

The study design, objectives, study population with patient inclusion/exclusion criteria, and the proposed research workflow are described in this section.

### 2.1. Study Design

**Figure 1:**
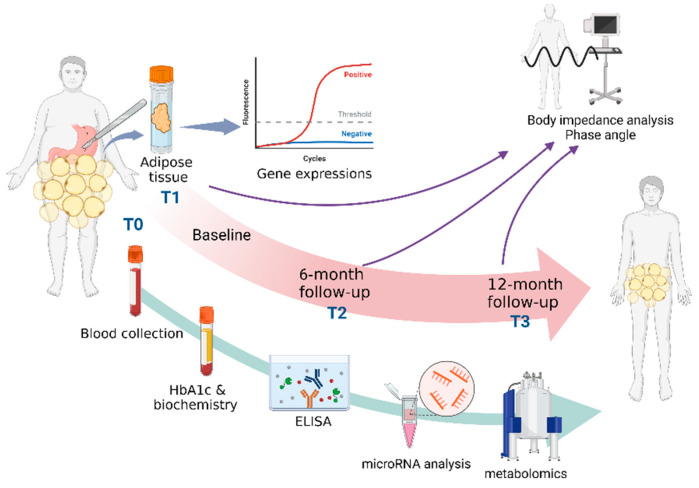
Overall design and methods used in the study. T0—baseline, pre-operative; T1—during surgery; T2—6-month follow-up; T3—12-month follow-up. Created with BioRender.com.

This is a multicenter prospective longitudinal study of patients with obesity and with different diabetes statuses who are scheduled for metabolic surgery at the Hospital Kuala Lumpur, iHeal Medical Centre KL, Sunway Medical Centre Selangor, Sunway Medical Centre Velocity KL, and KPJ Damansara Specialist Hospital, Malaysia. The two most standard metabolic surgical procedures performed at these hospitals are laparoscopic SGs and RYGBs. Serial measurements of the selected markers will be taken pre-operatively (baseline, T0) and at 6- (T2) and 12-month (T3) follow-ups after surgery. Visceral adipose tissue will be collected during surgery (T1). An overview of the scheduled sample collections for analysis is shown in Table 1. Figure 1 displays the design and methods that will be used in this study.

The objectives of this study are as follows:

(a) To study the effects of metabolic surgery on body mass index (BMI), body composition, and phase angle by comparing them pre- and post-metabolic surgery;
(b) To measure glycated hemoglobin (HbA1c), hormones, adipokines, and inflammatory cytokines, both pre- and post-metabolic surgery;
(c) To assess the level of miRNA, both pre- and post-metabolic surgery;
(d) To compare the gene expressions of visceral adipose tissue involved in inflammation, glucose, and lipid metabolism among different obese diabetes statuses;
(e) To determine the relationship between lifestyle and dietary practices, clinical outcomes, and the 1HNMR metabolic fingerprint.

The information obtained will be utilized to assist in the development of predictive biomarkers of T2D.

### 2.2. Study Setting

Patient recruitment: patients are being enrolled from the weight management centers and referred by endocrinologists from the obesity clinics. Surgeries and follow-up assessments will be conducted at the respective hospitals/medical centers. Blood samples will be collected for respective analysis. Hormones and adipokines measurement, molecular analysis, and 1H-NMR spectroscopy, with metabolomic analysis, will be conducted at the Institute for Medical Research.

### 2.3. Study Population

#### 2.3.1. Sample Size and Recruitment

This study will consist of three obese groups with different diabetes statuses who will be receiving metabolic surgery—SG or RYGB. The sample size was calculated using the G power statistical software (0.8 statistical power on a one-sided α-level = 0.05). Thirty-one patients need to be recruited for the study. We aim to include 34 patients in each group to allow for a loss of 10% in the follow-up rate.

Patient recruitment commenced in June 2022 and aimed to include 102 patients. The last follow-up is planned for June 2024. Subjects will be grouped into either non-diabetes, prediabetes, or T2D. The diagnostic criteria were based on the HbA1c values, defined in the Malaysia Clinical Practice Guidelines (2020) [20] (Table 2). Twenty healthy individuals will also be recruited as a control group for comparison.

#### 2.3.2. Patient and Healthy Individual Inclusion and Exclusion Criteria

Participants in the study are being recruited based on the inclusion and exclusion criteria outlined below.

##### Inclusion Criteria

The inclusion criteria for the subjects are as follows: age should be between 18 and 65 years; both men and women; body mass index (BMI) ≥25 kg/m^2^ (Asian BMI: WHO/IASO/IOTF (2000)) [21]; and scheduled for metabolic surgery. Obese patients who fulfill the criteria and have been diagnosed with prediabetes or T2D, according to their groups, are also eligible (Table 2). The inclusion criteria for healthy individuals are as follows: between 18 and 65 years of age; men and women; BMI between 18.5 and 22.9 kg/m^2^ (normal range BMI); and without prediabetes, diabetes, or any related comorbidities. All subjects must sign the written informed consent.

##### Exclusion Criteria

Patients or healthy individuals in the control group who use medications that may affect their body weight at the time they are recruited or who have other medical conditions—such as Cushing’s, acromegaly, heart failure, and Crohn’s disease—or who are alcohol-dependent will be excluded from the study. Individuals with a normal BMI of 18.5–22.9 kg/m^2^, but who present with comorbidities, will also be excluded.

### 2.4. Outcome Measures

**Figure 2:**
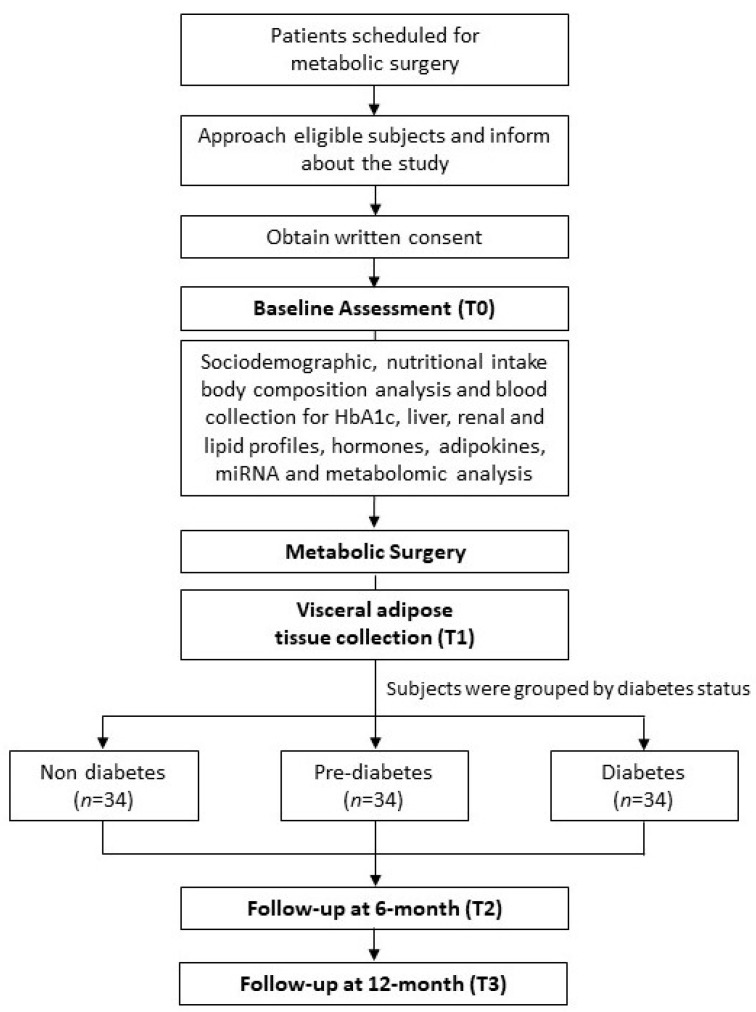
Proposed research workflow.

The outcome measures are primarily the anthropometric data; body composition analysis with PhA; biochemical parameters; miRNA levels; adipose tissue gene expressions; dietary patterns; and metabolomic profiles. After an overnight fast of 8–12 h, blood samples will be taken for HbA1c (glycated hemoglobin A1c), liver, renal, and lipid profiles, hormones, and adipokines, as well as metabolomic analysis. Later, miRNA will be extracted from the serum. Figure 2 shows the proposed research workflow.

### 2.5. Anthropometric Measurements, Clinical Assessment, and Laboratory Analysis

The clinical data and history (e.g., family health history of T2D, smoking, alcohol, and drug use) will be obtained from the medical records and interviews pre-operatively (T0). The follow-up assessments will be conducted at 6 ± 1 months (T2) and 12 ± 1 months (T3) after surgery. During the metabolic operation (T1), adipose tissue biopsies will be obtained for gene expression analyses. Refer to Table 1.

For anthropometric measurements, the body weight of the patient will be weighed to the nearest 0.1 kg and their height will be measured to the nearest 0.01 cm using a scale with a digital stadiometer, Solo Digital Clinical Scale (Detecto, MO, USA). The participants will wear light clothes and no shoes. Their BMI will be calculated and categorized according to the Asian BMI (WHO/IASO/IOTF, 2000) [21]. Their waist circumference will be measured using a SECA model 203 measuring tape (Seca GmbH & Co. KG, Hamburg, Germany). Body composition—especially body fat, visceral fat area, total body water, and fat-free mass—will be assessed using a multi-frequency bioelectrical impedance analyzer (BIA), and InBody S10 (Biospace, Seoul, Republic of Korea) will be used. The data input will include the patients’ age, sex, and height. The PhA values will be obtained directly from the BIA.

### 2.6. Biochemical Profile

Before blood collection, the subjects will be asked to fast overnight (8–12 h). An accredited laboratory will analyze routine blood biochemical parameters such as liver, lipid, and renal profile (Randox, Antrim, UK) and will be analyzed using a Dirui Chemistry analyzer (Dirui, Changchun, China). HbA1c will be measured using Tosoh G8 high-performance liquid chromatography (HPLC) analyzer (Tosoh, Tokyo, Japan) at the Endocrine and Metabolic Laboratory of the Institute for Medical Research. Hormones and adipokines—glucagon, adiponectin, apelin, omentin, ghrelin, glucagon-like-peptide-1 (GLP-1), C-peptide, interleukin (IL)-6, and leptin—will be analyzed using ELISA and multiplex assay analyses. The metabolomes will be explored via untargeted ^1^H Nuclear Magnetic Resonance (NMR) metabolomics, further explained in Section 2.10.

### 2.7. miRNA Analysis

Blood samples will be collected in a serum separator tube with separator gel. Next, they will be allowed to clot for 30 min, followed by 10 min of centrifugation at 2500× *g*. Immediately afterward, the serum samples will be stored at −80 °C until use. MicroRNA from the serum will be isolated using a miRNeasy Serum/Plasma (Qiagen, Hilden, Germany) following the manufacturer’s protocol. The Qiagen Serum/Plasma miRCURY LNA miRNA Focus PCR Panel (Qiagen, Hilden, Germany) will be utilized for profiling. We will perform the real-time PCR in a 96-well plate format using a StepOnePlus Real-Time PCR System (Applied Biosystems, Waltham, MA, USA), with SYBR green dye.

### 2.8. Adipose Tissue Gene Expressions

Samples of visceral adipose tissues will be collected during the surgery. The adipose tissue specimens will be placed in AllProtect Tissue Reagent (Qiagen, Hilden, Germany). Until further RNA extraction, the samples will be stored at − 80 °C.

Total RNA will be isolated from approximately 40 mg of tissue using an RNeasy Lipid Tissue Mini Kit (Qiagen, Hilden, Germany)., including QIAzol Lysis Reagent, as per the manufacturer’s protocol. The RNA’s quantity and quality will be assessed using a microvolume spectrophotometer (DeNovix 11 series) (DeNovix Inc, Wilmington, DE, USA). The expression of 84 genes related to the diabetes pathway will be analyzed using the Human RT2 Profiler PCR Array (SABiosciences, Frederick, MD, USA).

### 2.9. Dietary Patterns

Data on food consumption over the past month will be collected by the dietitian using a food frequency questionnaire (FFQ) that contains 14 food groups. This questionnaire was adapted from the Malaysian Adult Nutrition Survey [22]. The food portion sizes in the Malaysian Foods Album will be referred to for food portion calculation [23,24]. Overall, the steps for analysis will be based on established protocols [25,26,27].

### 2.10. Metabolomic Profile

Sample plasma will be prepared for nuclear magnetic resonance (NMR) metabolomic analysis according to published protocols [28]. Two milliliters of blood will be collected in lithium heparin tubes before centrifuging at 1500× *g* for 20 min. After dividing the plasma into aliquots, they will be stored at −80 °C until further analysis. The blood specimens will be collected in lithium heparin tubes for metabolomic study, because other anticoagulants, such as citrate and EDTA, produce additional high-intensity signals in the NMR spectrum [28,29].

After thawing the frozen plasma samples, 500 μL of the samples will be vortexed for one minute, followed by 2 min of 10,000× *g* centrifugation, to remove solid debris. A volume of 400 μL of plasma supernatant will be passed through a 0.5 ml, 3 kDa centrifugal filter (30 min at room temperature and 13,800× *g*) to remove macromolecules, such as lipids, proteins, lipoproteins, and the protein-bound forms of molecules [30]. The filters will be pre-washed three times with deionized water to eliminate the glycerol preservative [31]. To remove any leftover water, the filters will be inverted and centrifuged (13,800× *g* for 5 min). The ‘filtrates’ will then be transferred to another tube and diluted with phosphate buffer (KH_2_PO_4_) in deuterium oxide (D_2_O) containing 0.2% TSP and 0.1% imidazole in a 1:2 ratio. Each prepared sample of 600 μL will be transferred to a 5 mm NMR tube.

The 1D 1 H-NMR spectra will be collected at a temperature of 26 °C using a JNM-ECZ-600 R 600 MHz spectrometer (JEOL, Tokyo, Japan). The combination of PRESAT and the CPMG pulse sequence will suppress water signals and broad protein resonances [32]. After acquiring NMR spectra, they will be processed using the Chenomx NMR suite software, version 9.0 (Chenomx Inc., Edmonton, AB, Canada) [33,34,35]. The corresponding spectral data will be transformed into a table of standard integrals with a non-negative gvalue for multivariate data analysis (MVDA).

SIMCA-P software, version 17 (MKS Umetrics, Umea, Sweden), will be used to conduct MVDA on the processed spectra. First, the data will be mean-centered and Pareto-scaled by dividing each variable by its standard deviation’s square root. The spectral data will be analyzed using principal component analysis (PCA) and partial least-squares discriminant analysis (PLS-DA) [36,37,38,39]. To determine the validity of the PLS-DA model, a permutation test (100 cycles) and an ANOVA of cross-validated residuals (CVANOVA) will be performed. The prospective biomarkers in PLS-DA will be identified using loading plots with variable importance in the projection (VIP) values greater than one.

Using MVDA, Chenomx NMR suite version 9.0 software (Chenomx Inc., Edmonton, AB, Canada) will be used to identify and quantify different groups’ metabolites by identifying the sections of the spectrum (ppm) responsible. This will be accomplished by comparing the corresponding peak’s location, intensity, and linewidth with the 600 MHz HMDB metabolites library. The area under the peak will correspond to the metabolites’ relative concentrations [40].

The metabolic pathways’ identification will be conducted using the Kyoto Encyclopaedia of Genes and Genomes (KEGG) database with the MetaboAnalyst web application. Metabo-Analyst 5.0 (http://www.metaboanalyst.ca/) is a comprehensive tool suite for metabolomics data analysis [41] (Wieder et al., 2021). Currently, this platform offers 1600 pathways and visualizations for 21 model species, including rats, humans, mice, cows, and chickens.

## 3. Statistical Analysis

The quantitative variables will be expressed as the mean (standard deviation—SD). The Mann–Whitney test (quantitative variables) will be used to assess the differences among the groups. Spearman’s rho (ρ) test will be used to analyze the correlation between the quantitative variables.

The raw miRNA data will be preprocessed using the GeneGlobe Data Analysis Center (QIAGEN, geneglobe.qiagen.com). A Pearson correlation will be conducted to determine the relationship between the miRNAs and various clinical variables. This software will also be used for adipose tissue gene expression analysis. Research Electronic Data Capture (REDCap) will be used for data handling and statistical modeling in SPSS V.22. Statistical significance will be defined as *p* ≤ 0.05 in all analyses.

## 4. Discussion

Metabolic surgery no longer only includes mechanically restrictive and/or malabsorptive procedures, but also metabolic procedures that involve complex physiological changes [10]. These involve gut adaptation and influence the signaling pathways in several organs, such as the brain and liver, that regulate hunger, satiation and satiety, body weight, glucose metabolism, and immune functions [7,42]. Improvements have been noted after metabolic surgery, in which incretins, bile acids, and the microbiomes play their role in a complex interplay to improve metabolic health [43].

Metabolic surgery effectively achieves the remission of T2DM and other obesity-associated comorbidities [44,45,46,47]. Clinical trials involving bariatric surgery have shown that this surgery induces the remission of diabetes in 33–90% of individuals at one year post-surgery, compared with 0–39% of medically managed patients [48]. A study has highlighted a 50% prevalence of remission and a low prevalence of relapse following metabolic surgery. A meta-analysis has also explored the physiological mechanisms underlying T2D remission following metabolic surgery [49]. As we know, the remission of T2D following metabolic surgery is well established; however, identifying the patients who will go into remission is challenging [50]. However, the clinical data also reveal that remission is not achieved in every patient. To achieve diabetes remission among individuals, it is crucial to understand the determining factors that predict the glycemic response to surgery [48].

The amount of specific fat depots measured at follow-up is essential. Interestingly, whether or not a patient shows more significant long-term diabetes remission or incidence after bariatric surgery appears to be dependent on these fat depots [51]. The adipokines released, mainly by the visceral adipose tissue, will be monitored during the follow-ups in our study. Additionally, the visceral fat area will be observed as part of body composition measurement.

The phase angle (PhA) reflects the relationship between the resistance and reactance of the body, which is called impedance. It is considered a biological marker of cellular health. A high cell mass volume and potent cell membranes cause delayed signals, which results in a higher PhA. In critical illness states, a weakened membrane integrity, a reduced cell count, and a modified hydration status lead to a reduced PhA. These situations correlate with an increased mortality rate and the length of the hospital stay [52,53,54]. While many studies have been conducted on healthy subjects, obese individuals, the elderly population, and hemodialysis patients, we need more data on metabolic surgery diabetes remission patients [55,56].

miRNA is an epigenetic factor that regulates protein expression by destabilizing the target mRNA [57]. Owing to their accessibility—i.e., it does not require a very invasive procedure—the circulating miRNAs, which can be obtained from plasma or serum, could be used as biomarkers [58,59]. The miRNA levels can be determined by quantitative RT-PCR, which is straightforward, specific, and fast, with sensitive detection and quantification [60].

Dysfunctional adipose tissue is associated with the development of T2D and is the major source of circulating miRNAs. A specific miRNA, miR-122 is found explicitly in subcutaneous and visceral adipose tissues. The ratio of miR-122 in these tissues correlates with the outcome of bariatric surgery [61]. T2D remission prediction models have been designed for different surgery types. However, the model using miRNAs was only recently developed. In this model, the miRNAs are involved in obesity and insulin resistance [62]. Soon, the miRNA data could facilitate informed decisions about surgery.

Metabolomics shows excellent potential for extensively studying the metabolome’s dynamic alterations. In this prospective study, we will use NMR for untargeted analysis because it can uniquely identify and concurrently quantify a wide range of metabolomes. We expect to see changes in NMR profiling among obese diabetics who have undergone metabolic surgery—especially the metabolomes associated with energy homeostasis, alterations in lipid metabolism, and decreased branched-chain amino acids [63].

In the future, the liquid chromatography–mass spectrometry (LC–MS) technique will be used for targeted metabolomics analysis, because it is a powerful tool for analyzing polar metabolites in a complex sample [64,65]. Understanding how metabolic surgery affects diabetes will help to optimize its utilization for the disease’s prevention and treatment.

## Figures and Tables

**Table 1:** The time points of sample collection and clinical and laboratory analyses. T0 indicates preoperatively, T1 indicates during operation, T2 indicates 6 ± 1 months after the operation, and T3 indicates 12 ± 1 months after the operation.

Item	T0	T1	T2	T3	Outcomes
Medical history and anthropometric measurements	X		X	X	Age, gender, ethnicity, weight, and height.
Bioimpedance analysis	X		X	X	Percentage body fat, visceral fat area, skeletal muscle mass, body water content, and phase angle.
Blood collection	X		X	X	Glycated hemoglobin (HbA1c), lipid, renal, and liver profiles. Hormones and adipokines, miRNA (serum), and metabolomics (plasma).
Adipose tissue biopsy		X			RNA for gene expression study.
Diet record	X		X	X	Dietary patterns

**Table 2:** Diagnostic values for prediabetes and type 2 diabetes (T2D) based on HbA1c values.

Category	HbA1c (%)
Normal	<5.7
Prediabetes	5.7–<6.3
Type 2 diabetes (T2D)	≥6.3

## Data Availability

Not applicable.
